# Homœopathic Treatment of Toothache from Pulpcapping

**Published:** 1894-11

**Authors:** Charles H. Taft

**Affiliations:** Chicago, Ill.


ARTICLE V.

### HOMEOPATHIC TREATMENT OF TOOTHACHE FROM PULP-CAPPING.

CHARLES H. TAFT, D. M. D., CHICAGO, ILL.

The interest which has been awakened in the homeopathic treatment of cases occurring in the every-day practice of the dentist, as manifested by the numerous letters I have received requesting such information as I could give and which would enable others to pursue a line of study intelligently, with the end in view of having at command, if nothing more, an effectual method of quickly relieving the various kinds of toothache, from causes with which we are all familiar, prompts me to relate a recent case in practice, and to suggest some of the indications which furnish a key-note for the remedy given.

As a preface to the subject, I may say that in recent papers I have dwelt at some length on the philosophy of the homeopathic law of cure, and have suggested the work entitled "Hering's Domestic Physician," with its chapter on the affections of the teeth, as furnishing, to me at least, an invaluable aid for the treatment of many cases where the ordinary dental therapeutic agents at our command have proved unsuccessful.

A complete mastery of the art of healing, or the ability to relieve human suffering, even when based on a simple and well-established law, is not to be acquired in a day, if indeed in a lifetime; but to the earnest and thoughtful student, neophyte though he be, there is a fascination in the study of this law and the manifestations of its operation which cannot fail to urge one on to still greater effort, when once such study has been entered on free from partisan prejudice and with the simple desire to find the truth.

I am well aware that many practitioners make it a practice never to cap an exposed pulp, even though the pulp is a freshly exposed one and devoid of any inflammation or irritation, but deem it best to destroy it at once. I destroy it only when I am confident its vitality cannot be successfully restored and maintained without giving the patient future and continual annoyance; for I am of the opinion that a live tooth, like any other organ, is an infinitely better member of the economy than is one in which such vitality has been destroyed, and especially if the tooth happens to be one of the incisors or bicuspids.

The following case in practice is intended to furnish one of the many reasons for such belief, and to suggest such therapeutic treatment as a similar emergency may demand.

On March 16th, Mr. F., aged thirty-two, had a large corono-distal cavity in the right upper second bicuspid, and another in the corono-mesial surface of the right upper first molar. They were carefully excavated, but not without avoiding an exposure of the pulp in the molar, and perilously near one in the bicuspid. Both teeth were exquisitely sensitive to the touch of an excavator, but had never given the patient the slightest discomfort. The exposed pulp was capped by applying oil of cloves to the pulp and dusting over it dry oxid of zinc. The same precaution was taken with the bicuspid, and both cavities were then filled with oxiphosphate, mixed to the proper consistency. The operation finished, the rubber-dam was removed; the patient felt no pain or discomfort from the fillings, and, rinsing out the mouth, was about to leave the chair, when he seized his head with both hands and cried aloud with pain.

The pain continued for several minutes, coming in terrific paroxysms of but a few seconds apart, with determination of blood to the head and face. Becoming well-nigh frantic with pain, he implored me to extract the tooth at once, and in no uncertain tone, saying that he simply could not and would not stand it.

Watching his symptoms for a few moments, I placed on his tongue a few pellets of the potentized chamomilla, and waited for its effect.

Between the paroxysms he asked what I had given him, and, contrary to my usual custom of naming the remedy, I said it was chamomilla.

With a good knowledge of remedies, being in the wholesale drug business, he remarked with no apparent confidence in me or the remedy, that if chamomilla stopped such suffering as his, it would be the first time he ever heard of its so doing. He added, however, before he left the office, that he was open to conviction.

As I stood by the chair, watching him in his writhing, I feel sure I had made no mistake in the selection of the remedy, and that, like an arrow from the bow, it would go straight to the mark. Within five minutes his writhing ceased, and the pain melted away like dew before the sun; his face began quickly to assume its normal color, and the paroxysms only recurred at long intervals and with decreasing severity during the next fifteen minutes, till they ceased entirely. He remained an hour after the medicine had taken effect, fully expecting the pain to return and compel me to extract the tooth, but it was obstinate and positively refused to ache. Finally he left the office, with the assurance from me that in all probability there would be no recurrence of pain, and with an equally strong belief on his part that he would have to hunt up a dentist in the middle of the night and have his tooth extracted.

Up to the present writing, during which time the patient has had several subsequent sittings, there has been not the slightest recurrence of pain or discomfort.

Doubtless some skeptical friend will venture the remark that the cessation of pain was not caused by the medicine, but ceased of its own accord. Another will probably suggest that I hypnotized him. A third will emphatically assert it was not the medicine, for how could attenuated "moonshine" effect so complete a relief? While a fourth will incline to the belief that I employed the methods of the Christian Scientist, and assured him that his suffering was simply a delusion and did not really exist.

To all such my statement will undoubtedly be of little interest or help in similar cases, but to those who are interested in the action of remedies and the laws which govern them, so far as it concerns us in our every-day practice, this paper is especially addressed, with a view of pointing out the fact that one of the strongest indications of chamomilla for the relief of a toothache like the one described is the fact of its driving the patients so nearly frantic with pain that it is difficult for them to speak a pleasant word, or in a pleasant tone of voice. In a word, are thoroughly ugly.

In striking contrast to the always jovial, happy-go-lucky disposition of the patient, was his manner and language toward me during those first fifteen minutes after the operation. That and the sudden rush of blood to the cheeks were two of the strongest indications pointing to chamomilla in preference to other drugs having equally strong indications in other cases, but which, being not homœopathic, would have proved equally unavailing and unsuccessful.

On the ability to differentiate clearly between the symptoms in any given case like the above, which plainly point to one remedy, and only one, depends the success of him who attempts to apply the art of dental medicine in strict accordance with the law of homœopathy or similars.

It was Samuel Hahnemann who said:

"When we have to do with an art whose end is the saving of human life, any neglect to make ourselves thorough masters of it becomes a crime."

When we, as dentists, have to do with an art one of whose ends is the alleviation of human suffering, should a neglect to make ourselves thorough masters of it become any the less a crime? The question is one that may well command our thoughtful consideration, and must be answered by every man according to his own best judgment and with such methods as he alone has at his command.—*International.*

